# Early Detection of Acute Drug-Induced Liver Injury in Mice by Noninvasive Near-Infrared Fluorescence Imaging

**DOI:** 10.1124/jpet.116.238378

**Published:** 2017-04

**Authors:** Kristine O. Vasquez, Jeffrey D. Peterson

**Affiliations:** PerkinElmer Inc., Hopkinton, Massachusetts

## Abstract

Hepatocellular and cholestatic forms of drug-induced liver injury (DILI) are major reasons for late-stage termination of small-molecule drug discovery research projects. Biochemical serum markers are limited in their ability to sensitively and specifically detect both of these common DILI forms in preclinical models, and tissue-specific approaches to assessing this are labor intensive, requiring extensive animal dosing, tissue preparation, and pathology assessment. In vivo fluorescent imaging offers noninvasive detection of biologic changes detected directly in the livers of living animals. Three different near-infrared fluorescent imaging probes, specific for cell death (Annexin-Vivo 750), matrix metalloproteases (MMPSense 750 FAST), and transferrin receptor (Transferrin-Vivo 750) were used to measure the effects of single bolus intraperitoneal doses of four different chemical agents known to induce liver injury. Hepatocellular injury–inducing agents, thioacetamide and acetaminophen, showed optimal injury detection with probe injection at 18–24 hours, the liver cholestasis-inducing drug rifampicin required early probe injection (2 hours), and chlorpromazine, which induces mixed hepatocellular/cholestatic injury, showed injury with both early and late injection. Different patterns of liver responses were seen among these different imaging probes, and no one probe detected injury by all four compounds. By using a cocktail of these three near-infrared fluorescent imaging probes, all labeled with 750-nm fluorophores, each of the four different DILI agents induced comparable tissue injury within the liver region, as assessed by epifluorescence imaging. A strategy of probe cocktail injection in separate cohorts at 2 hours and at 20–24 hours allowed the effective detection of drugs with either early- or late-onset injury.

## Introduction

Drug-induced liver injury (DILI) is the leading reason for termination of drug discovery research projects, is a significant concern in attrition of new drug molecules reaching phase III clinical trials, is the major reason for US Food and Drug Administration withdrawal of drugs from the market after approval, and accounts for more than 50% of acute liver failure cases in the United States (Bissell et al., 2001). Toxicity-related attrition in drug development can occur at all stages of the preclinical research process, with later-stage identification of toxicity obviously being the most costly; therefore, early elimination of hepatotoxicity-inducing compounds during drug discovery and preclinical development is an important priority.

One new tactic in toxicology is to focus on identifying perturbations of relevant biomarkers associated with important biologic functions and pathways (Gibb, 2008; Bhattacharya et al., 2011; Krewski et al., 2011). This could offer some potential advantages over conventional toxicology approaches in both sensitivity and ease of application. Depending on the drug being tested and its potential toxic mechanism(s), a variety of different biologic pathway changes can occur in liver tissue, including those indicating direct hepatocyte injury (apoptosis/necrosis), oxidative stress, vascular or microvascular injury, induction of innate or cognate immunity/inflammation, and DNA damage. Within each of these mechanisms are multiple signaling pathways and biomarkers that could serve as indicators of drug effects; therefore, a strategy of pathway/biomarker profiling has the potential for the sensitive detection of drug-induced changes for early-stage screening. Generally, this systems-based toxicology strategy utilizes in vitro experimental models and addresses dozens of biomarkers from interconnected or parallel networks; however, our hypothesis was that such an approach could be adapted for in vivo use in a scaled-down fashion by using simultaneous detection of a few key biomarker responses. This should retain the concept of screening for different types of adverse biologic responses, rather than relying on grosser tissue phenotypic changes, but opens up the possibility of using noninvasive rodent imaging strategies.

Recent advances in optical imaging and near-infrared (NIR) probes (Weissleder and Ntziachristos, 2003; Korideck and Peterson, 2009; Krautz-Peterson et al., 2009; Kossodo et al., 2010; Peterson et al., 2010; Bao et al., 2012; Lin et al., 2012; Zhang et al., 2012; Eaton et al., 2013; Daghighi et al., 2014) have shown the benefit of biologic, rather than phenotypic or anatomic, readouts in preclinical drug efficacy research. Although imaging of preclinical models of safety/toxicology has not been pursued extensively to date, the ability to use fluorescent imaging probes that detect and quantify a variety of biologic activities has considerable potential in this area as well (Amoozegar et al., 2012; Shuhendler et al., 2014; Peterson, 2016). In this study, three different types of NIR fluorescent probes—Annexin-Vivo 750 (AV-750), MMPSense 645 or 750 (MMP-645 or MMP-750), and Transferrin-Vivo 750 (TfV-750), specific for cell death, matrix metalloprotease (MMP) activity (inflammation), and transferrin receptor (metabolic activity) expression, respectively—were found to be excellent tools for detecting/characterizing drug-induced tissue injury in general and DILI in particular. A single dose of well characterized hepatocellular injury–inducing agents thioacetamide (TAA; 300 mg/kg) or acetaminophen (APAP; 300 mg/kg) showed late induction of acute injury (optimal probe injection, approximately 18–22 hours), the liver cholestasis-inducing drug rifampicin (RMP; 300 mg/kg) showed a very early liver response (optimal probe injection, approximately 2 hours), and chlorpromazine (CPZ; 100 mg/kg), which induces mixed hepatocellular/cholestatic injury, showed both early and late signals, with different patterns of liver responses among these different probes seen for the four different chemical agents. By using a cocktail of these three NIR fluorescent imaging probes (AMT-750), all labeled with 750-nm fluorophores, each of the four different chemical agents showed a comparable total fluorescent signal within the liver region by epifluorescence imaging, offering a potentially useful universal DILI imaging strategy to be applied to compounds of unknown DILI potential or mechanism. The use of AngioSense 680 (AS-680), in combination with the AMT-750 cocktail of probes, provided further information regarding drug-induced vascular changes and allowed correction for any contribution of passive accumulation of the 750-nm probe cocktail. A strategy of probe injection in separate cohorts at 2 hours and at 20–24 hours allowed the sensitive identification of chemical agents with either early- or late-onset injury to limit the possibility of false negative results. Compared with conventional plasma/serum assays, in vivo imaging can offer fast, quantitative imaging results that directly assess the tissue of interest. Our results to date demonstrate the potential of optical imaging to assess possible drug-induced liver toxicity early in drug discovery programs.

## Materials and Methods

### 

#### Experimental Animals.

For drug-induced liver toxicity studies, male BALB/c mice (aged 7–10 weeks) and C57BL/6 mice (aged 7 to 8 weeks to minimize skin pigment interference after 8 weeks) were obtained from Charles River Laboratories (Wilmington, MA) and maintained in a controlled environment (72°F; 12-hour/12-hour light/dark cycle) under specific pathogen-free conditions with water and low fluorescence chow (Envigo, Cambridgeshire, UK) provided ad libitum. C57BL/6 mice induced with APAP were fasted overnight (approximately 18 hours) prior to drug administration. For this particular treatment, the use of C57BL/6 mice was essential, as well as the use of males; female C57BL/6 mice and mice of other strains show low liver toxicity under the conditions explored in this research (Duan et al., 2016). For all other treatments, male BALB/c mice were used. All experiments were performed in accordance with recommendations in the National Institutes of Health Guide for the Care and Use of Laboratory Animals. The protocol (04-0512) was approved by PerkinElmer’s Institutional Animal Care and Use Committee guidelines. No invasive or surgical procedures were used in these studies, but all imaging activities were performed under appropriate anesthesia to minimize animal distress.

#### Fluorescent Probes for the Detection of Tissue Injury.

Four commercially available NIR fluorescent imaging probes (PerkinElmer Inc., Hopkinton, MA) were used to detect drug-induced tissue injury. AV-750 (fluorophore-labeled Annexin V protein) was used to detect apoptosis and early necrosis in affected tissues. MMP-645 and MMP-750 probes (fluorophore-labeled MMP substrate peptide [P-L-G-V-R]), pan-specific for the MMP family of enzymes, were used to detect secretion of these proteases by inflammatory cells. The TfV-750 probe (fluorophore-labeled transferrin protein) was used to detect changes in transferrin receptor expression indicative of metabolic changes within the liver. AS-680 is a vascular probe designed to detect blood leakage into tissue either due to edema or vascular injury. For studies in which individual imaging probes were used, the standard recommended dose from the product insert was used. Fluorescence was measured at the following recommended time points: 24 hours post-injection for all probes except for AV-750, which was measured at 2 hours (with the signal still present at 24 hours). For studies involving a cocktail of imaging probes (AMT-750), a 3:8:1 ratio was used (AV-750/MMP-750/TfV-750; i.e., 1.5 nmol AV-750 was combined with 4 nmol MMP-750, and 0.5 nmol TfV-750 for each mouse injection). This maximized the specific drug-induced signal while minimizing the background liver and kidney signals. AMT-750 imaging was performed 24 hours after probe injection. The standard dose of 2 nmol AS-680 per mouse was added to the imaging cocktail in the final imaging studies.

A variety of other NIR fluorescent imaging probes are available that capture different molecular aspects of fibrosis, inflammation, calcification, glomerular filtration rate, and gastric-emptying rate associated with drug-induced liver, vascular, kidney, and gastric tissue damage (Table 1).

**TABLE 1:** Toxicology tissue changes and NIR imaging probes

Toxicology/Tissue	Biologic Tissue Response	Potential Imaging Probe
General markers	Inflammation	MMPSense 680 and 750 FAST*^a^*
		ProSense 680, 750EX, and 750 FAST
		Neutrophil Elastase 680 FAST
	Apoptosis/necrosis	Annexin-Vivo 750*^a^*
	Vascular changes	AngioSense 680EX and 750EX*^a^*
Liver specific	Metabolism	Transferrin-Vivo 750*^a^*
Kidney specific	Glomerular filtration	GFR-Vivo 680*^a^*
	Renin-angiotensin system	ReninSense 680 FAST
Stomach specific	Gastric emptying	GastroSense 680*^a^*
Soft tissue calcification	Calcium deposition	OsteoSense 680 and 750*^a^*
	Cathepsin K activity	Cat K 680 FAST*^a^*

^a^ Probes that are commercially available with some validation data for use in preclinical drug-induced tissue injury applications (Peterson, 2016).

#### DILI Studies.

A panel of small chemical compounds with established toxicity profiles were used in acute, single-dose studies in BALB/c or C57BL/6 mice. TAA is an organosulfur compound that has been used as a substitute for hydrogen sulfide, as a stabilizer of motor fuel, and as a topical preventative for mold growth on fruit. It is known as a class 2B carcinogen that also induces marked hepatocellular toxicity in animals (Wang et al., 2004; Ackerman et al., 2015). RMP is a widely used antimicrobial agent that is a crucial component in treatment regimens for tuberculosis. This drug causes hepato- and nephrotoxicity as well as abdominal cramps and stomach distension (Zítková et al., 1982; Katz and Lor, 1986; Zargar et al., 1990; Tassaduq et al., 2011). CPZ is widely used as a sedative or antiemetic and is also one of the primary antipsychotic medications used to treat schizophrenia. This drug is known to induce acute inflammation-mediated cholestatic liver injury (Mullock et al., 1983) and changes in kidney function (Berg and Bergan, 1976). APAP is an over-the-counter medication that is widely used for treatment of pain and fever. Acute overdoses are well known to cause rapid, potentially fatal, hepatocellular injury, which is the leading cause of acute liver failure in the Western world due to either deliberate overdose, accidental overdose, or other risk factors with therapeutic dosing (Maddox et al., 2010; Ging et al., 2016; Yoon et al., 2016). This drug induces marked hepatocellular toxicity in C57BL/6 mice with significant variations between mouse strains (McGill et al., 2012; Duan et al., 2016).

APAP (catalog no. A7085; Sigma-Aldrich, St. Louis, MO) was freshly prepared in phosphate-buffered saline at a concentration of 20 mg/ml. The phosphate-buffered saline was preheated in a 55°C water bath to facilitate dissolving the powder. RMP (catalog no. R3501; Sigma-Aldrich) was prepared in a complexing agent, (2-hydroxypropyl)-*β*-cyclodextrin solution (catalog no. H5784; Sigma-Aldrich), at a concentration of 10 mg/ml. CPZ (catalog no. C8138) and TAA (catalog no. 163678; both from Sigma-Aldrich) were each prepared in water at a concentration of 20 mg/ml.

Mice were injected intraperitoneally with chemical compounds as a single bolus dose and injected at different times post-treatment with NIR fluorescent imaging probes or probe cocktail. Immediately prior to imaging, mice were anesthetized using isoflurane inhalation and were depilated to minimize fur interference with the fluorescent signal. Nair lotion (Church and Dwight Co., Inc., Princeton, NJ) was applied thickly on the skin over the torso (front, back, and sides) of each mouse, rinsed off with warm water, and reapplied until all fur was removed. Imaging was performed on the IVIS SpectrumCT imaging system (PerkinElmer, Inc.) by two-dimensional (2D) epifluorescence imaging at indicated times after probe injection to optimize readouts for DILI. In preliminary studies, mice were injected intraperitoneally with a range of doses of liver injury–causing compounds, from 30 to 600 mg/kg (data not shown). From these data, a dose of 300 mg/kg was selected for RMP, APAP, and TAA, and a lower dose of 100 mg/kg was chosen for CPZ to minimize excessive vascular damage caused at higher doses. BALB/c male mice were used for all compounds except for APAP, which is known to require C57BL/6 male mice for optimal elicitation of liver injury.

#### Ex Vivo Serum Alanine Transaminase and Alkaline Phosphatase Assessment.

Serum was collected from BALB/c mice treated with single intraperitoneal doses of CPZ (100 mg/kg), RMP (300 mg/kg), TAA (100 mg/kg), and APAP (500 mg/kg) 24 hours after dosing. Samples were assayed for alanine transaminase (ALT) and alkaline phosphatase (ALP) (QuantiChrom Alkaline Phosphatase Assay Kit and EnzyChrom Alanine Transaminase Assay Kit, respectively; both from BioAssay Systems, Hayward, CA) using the manufacturer’s protocols.

#### Ex Vivo Fluorescence Assessment of Affected Tissues.

After animals were imaged in vivo, they were euthanized by carbon dioxide asphyxiation. The organs (brain, heart, lungs, liver, pancreas, spleen, stomach, intestines, kidneys, fat, and skin) were removed postmortem. Epifluorescence images of the organs were acquired, followed by tissue fixation in 10% formalin.

#### Histopathology.

A select set of tissues (liver, brain, lung, heart, stomach, skin, fat, spleen, pancreas, intestine, and kidney) were fixed in 10% neutral buffered formalin for 24 hours at 20°C, followed by storage in 70% ethanol. Tissues were then embedded in paraffin, sectioned (4 *µ*m), and stained with hematoxylin and eosin for general evaluation of pathologic changes.

#### Statistical Analysis.

Data are presented as means ± S.E.M. Analysis of significance was conducted using analysis of variance with the Dunnett post-test in comparison with untreated control animals/tissues. *P* < 0.05 was considered significant.

## Results

### 

#### Modes of Optical Imaging for Detection and Quantification of Liver Fluorescence.

**Fig. 1.**
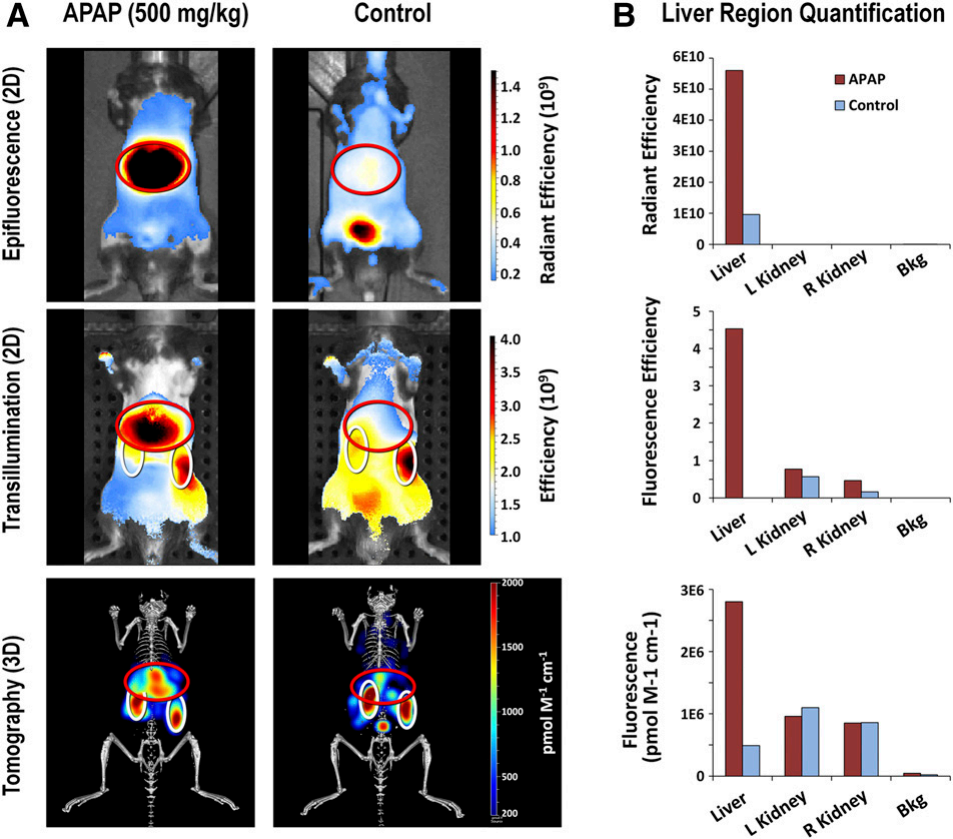
Comparison of three optical imaging strategies for detecting liver fluorescence. APAP-treated (500 mg/kg) and control male C57BL/6 mice were injected 24 hours later with AV-750 and fluorescent images were acquired 2 hours later on the IVIS SpectrumCT. (A) Representative images are shown of one treated animal and one control animal by 2D epifluorescence (upper panel), 2D transillumination fluorescence (middle panel), and 3D fluorescence tomography (lower panel), and ROIs were placed to capture the signal in the liver and kidneys. Control region ROIs (not shown) were placed in the depilated right flank region for background subtraction. (B) Quantification of the liver and kidney AV-750 fluorescent signal was determined in treated and control mice for each of the fluorescent imaging approaches. Bkg, background control region; L, left; R, right; ROI, region of interest.

A unique approach to liver injury measurement, using fluorescence imaging, was assessed that could provide a means for rapid screening of liver biology changes, rather than looking for overt tissue changes, using minimal compound in a single high-dose bolus-injection model in mice. One of the most obvious biologic responses to explore as a readout was cell death, so we used an Annexin V–based NIR fluorescent imaging probe (AV-750) that has been used effectively in cancer treatment research (Zhao et al., 2015) and in preliminary liver injury studies (Peterson, 2016). Such a probe should be useful for assessing drugs known to induce hepatocellular liver toxicity that involves direct induction of cytotoxic liver apoptosis/necrosis. To assess whether optical imaging can be used to detect acute drug-induced damage in the liver, a variation of a well established mouse model of DILI was used: male C57BL/6 mice were injected intraperitoneally with a single bolus dose of 500 mg/kg APAP followed 24 hours later by intravenous injection of the NIR fluorescent AV-750 imaging probe to detect apoptosis/necrosis in the liver. Three modes of optical imaging were used (Fig. 1): 1) 2D epifluorescence imaging, 2) 2D transillumination fluorescence imaging, and 3) three-dimensional (3D) tomographic imaging. Epifluorescence provided a rapid and robust approach for imaging five animals at a time, although this approach was limited in its ability to detect the deep tissue signal. Transillumination (2D) imaging used a moving point light source placed behind the subject, yielding a composite image that revealed a blending of the deep tissue and superficial signals. Tomography (3D) used a similar transillumination approach, but rather than blending the results in a 2D representation, the results from multiple image acquisitions were run through a mathematical algorithm to model 3D localization within the body of the subject. All three approaches were highly effective in detecting increases in AV-750 liver binding induced by APAP (Fig. 1A). Tomography was the most useful approach for detecting and discriminating the liver and kidney signals; however, it was limited to one mouse at a time and required approximately 20 minutes per mouse. Epifluorescence imaging offered the best combination of rapid multiple animal imaging and sensitive detection of the liver signal. The 2D transillumination approach was least effective, in that image acquisition was relatively slow and the final results yielded kidney signal overlap with the analysis of liver fluorescence. Quantitatively, all three approaches effectively showed an increased AV-750 signal compared with control mice (Fig. 1B). Epifluorescence and tomographic approaches correlated well with each other, and both correlated well with quantification of the 2D epifluorescence signal in liver tissues ex vivo (data not shown).

#### Epifluorescence Profiling of Three NIR Fluorescent Imaging Probes in Mice Treated with Four Different Liver Injury–Inducing Chemical Agents.

Preliminary studies using AV-750 to assess different DILI-inducing chemical agents suggested that this probe is a sensitive detector of hepatocellular toxicity (Peterson, 2016) but may not be as effective in detecting other forms of liver injury such as cholestasis. In part, this may be due to our stringent requirement that our imaging system/probes detect liver changes after only a single compound administration, and this could be attributed, in part, to the additional mechanistic complexity in other forms of DILI in which additional time may be needed for inflammation or bile duct blockage events to occur.

Other biomarkers, for which there are imaging probes, are known to change in expression during different types of liver injury. For example, iron is an essential nutrient that is tightly regulated by the liver, and excessive iron uptake is one of the major mechanisms contributing to increased steatohepatitis, fibrosis, and cirrhosis (Anderson and Shah, 2013). Transferrin is generated in the liver as the major serum iron-binding protein in the body, and both transferrin (Amacher et al., 2005) and the transferrin receptor (Cairo and Pietrangelo, 1995; Izawa et al., 2014) were found to change expression during chemically induced liver injury. MMPs have also been implicated as biomarkers of liver injury, as indicators of both inflammation and fibrosis. In particular, MMP-2 and MMP-9 have been shown to play a role in CCl_4_-induced hepatic fibrosis (Domitrović et al., 2013; Al-Olayan et al., 2014). To assess the potential benefit of combined screening for cell death, MMP activity, and transferrin receptor expression on the detection of DILI, we used fluorescent imaging probes specific for these biomarkers (AV-750, MMP-645, and TfV-750, respectively) to image the livers of animals receiving four different liver injury–inducing chemical compounds. Two of these compounds (TAA and APAP) are known to induce hepatocellular injury (McGill et al., 2012; Ackerman et al., 2015), a third drug (RMP) predominantly induces liver cholestasis (Zítková et al., 1982; Katz and Lor, 1986; Zargar et al., 1990), and a fourth drug (CPZ) induces mixed hepatocellular/cholestasis injury (Mullock et al., 1983). Serum ALT/ALP ratios can provide a rough mechanistic assessment, with hepatocellular injury defined as a ratio > 5, cholestasis as a ratio < 2, and mixed hepatocellular/cholestasis with a ratio between 2 and 5. Our serum ALT/ALP results (from mice receiving single intraperitoneal bolus doses of chemical compounds) agreed well with previous publications regarding mechanistic interpretation of each of the chemical agents: ALT/ALP ratios were > 15 for APAP (500 mg/kg) and TAA (100 mg/kg), 0.5 for RMP (300 mg/kg), and 1.8 for CPZ (100 mg/kg).

**Fig. 2.**
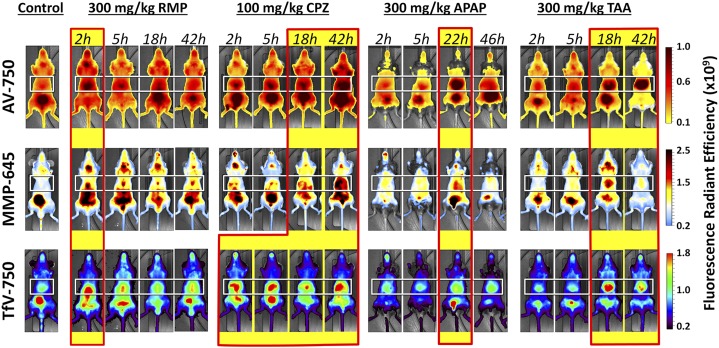
Assessment of four chemical compounds and three imaging probes in liver injury imaging. Whole-mouse ventral epifluorescence imaging was used to detect accumulation of AV-750, MMP-645, and TfV-750 in the liver regions of different cohorts of mice at different times post-treatment. Mice (*n* = 3 per group) were injected intraperitoneally with the indicated drug doses and then injected intravenously with imaging probes at the indicated times (2, 5, 18/22, and 42 hours). Whole-body epifluorescence images were acquired on the IVIS SpectrumCT 2 hours after AV-750 treatment or 24 hours after MMP-645 and TfV-750 treatment. Representative individual mice are shown for each drug/probe combination, with the same mouse represented longitudinally, and this experiment is representative of three independent studies. White boxes indicate the liver regions quantified, and red-outlined yellow boxes show the optimal probe injection times post-treatment.

**Fig. 3.**
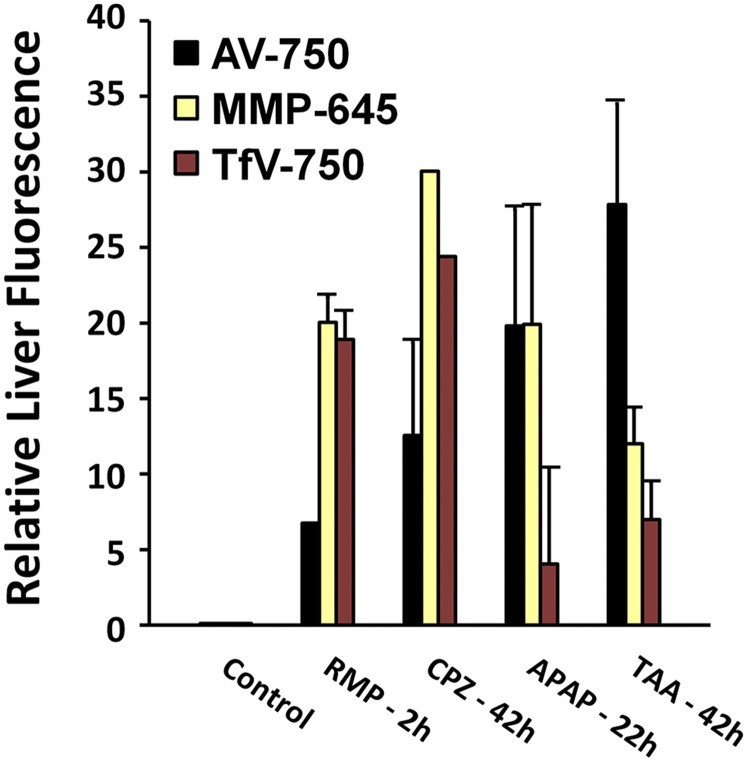
Multiple fluorescent probe profiling in the liver response to chemical insult. Quantification of the liver signal from noninvasive imaging in Fig. 2 was determined using 2D region-of-interest placement positioned to capture and quantify the fluorescent signal in the liver region. Results are represented for the optimal probe/time point conditions as fold above background after standard 90% background thresholding to best illustrate profile trends for the different treatments.

Whole-mouse epifluorescence imaging (Fig. 2) was used to detect accumulation of AV-750, MMP-645, and TfV-750 in the liver regions of different cohorts of mice by injecting these probes at different times after treatment (2, 5, 18–22, and 42 hours). AV-750 was imaged 2 hours after probe injection, and the other two probes were imaged 24 hours after probe injection to achieve optimal probe performance with regard to the peak signal and the maximal washout of unbound probe. In all cases, based on pharmacokinetics and biodistribution, the biologic changes detected likely occurred within a 0- to 10-hour time frame relative to probe injection, even in instances in which actual images were acquired at 24 hours. The apparent kinetics of liver change differed between the different chemical agents and the different probes. Interestingly, earlier changes were associated with those chemical agents known to induce cholestasis (i.e., RMP and CPZ), whereas later changes were associated with chemically induced hepatocellular damage (i.e., APAP and TAA). CPZ, which is known to induce mixed cholestasis/hepatocellular liver injury, showed both an early and late liver signal. Although AV-750 was optimally imaged 2 hours post-injection, additional studies indicated good probe retention in the liver up to 24 hours (data not shown), allowing the possibility of aligning imaging time points between different probes more easily. Quantification of results for each of these chemical agent/probe combinations (Fig. 3) was performed in datasets representing the peak response, which was 2 hours (post-treatment) for RMP, 42 hours for CPZ, 22 hours for APAP, and 42 hours for TAA. Numbers reflect fold increases over background after threshold correction for the background signal to best adjust to absolute magnitude differences attributable to both probe and fluorophore wavelength. RMP CPZ showed dominant MMP-645 and TfV-750 signals, and the two hepatocellular toxicity–inducing chemical agents (APAP and TAA) were generally dominated by AV-750 signal.

#### Testing of NIR Fluorescent Three-Probe Cocktail for General Liver Injury Imaging.

**Fig. 4.**
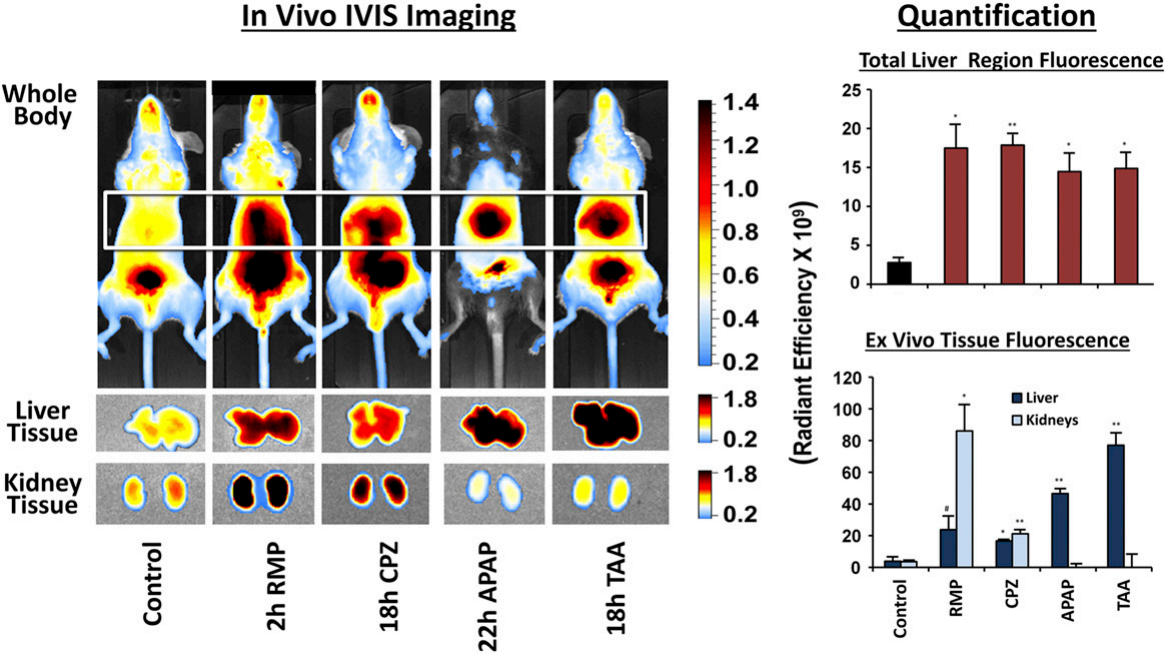
Validation of a three-probe fluorescent cocktail for general detection of DILI. A cocktail of three imaging probes (AV-750, MMP-750, and TfV-750) was optimized for 24-hour imaging in mice treated with all four drugs (*n* = 3 per group). Drug-dosed mice were injected with the imaging cocktail at two different times post-treatment (18 hours for TAA and APAP; 2 hours for CPZ and RMP), and all mice were imaged 24 hours after AMT-750 for both noninvasive liver assessment (upper left panel) and ex vivo tissues (lower left panel). Epifluorescence images of mice (left panel) of mice receiving optimal doses of RMP, CPZ, APAP, or TAA show liver region signal in comparison with control mice. Quantification of the liver signal from noninvasive imaging (upper right panel) and the ex vivo liver and kidney signal (lower right panel) were determined by region-of-interest placement to capture the entire liver or individual tissues, and results were represented as the total liver fluorescent signal ± S.E. Statistical significance was assessed by analysis of variance with the Dunnett post-test (*P < 0.01; **P < 0.001; ^#^*P* < 0.05; *n* = 3). Results are representative of multiple studies using either multiplex or individual probe imaging.

The effectiveness of AV-750, MMP-645, and TfV-750, each showing some level of detection of liver injury induced by four different chemical agents, raised the possibility of using them in combination. One strategy would be to use them each on a different spectral channel, with triplex imaging providing three independent datasets. Alternatively, one could pursue a simpler approach by combining all three probes as a cocktail in the same spectral channel. The second approach fits well with our overall concept of a simple screening paradigm, used early in the drug discovery process, that requires only small amounts of compounds and small numbers of mice. A cocktail of these imaging probes was generated from 1.5 nmol AV-750 per mouse, 4 nmol MMP-750 per mouse, and 0.5 nmol TfV-750 per mouse based on preliminary studies designed to maximize the liver signal while minimizing the normal background liver/kidney signal (data not shown). This cocktail of probes (AMT-750) was tested in mice receiving single bolus doses of RMP, CPZ, APAP, or TAA; AMT-750 was injected at appropriate time points after chemical agent bolus (based on achieving the maximal AV-750 signal), and imaging was performed 24 hours later. All four treatment groups showed clear, liver region fluorescence by 2D epifluorescence imaging, and signals were statistically significant compared with control animals, ranging from 5- to 7-fold increases (Fig. 4). Fluorescence increases were comparable with the different chemical agents, suggesting that the AMT-750 cocktail effectively and equivalently detected hepatocellular injury, cholestasis, and mixed liver injury in mice. Ex vivo liver tissue imaging confirmed the apparent liver changes seen noninvasively; however, other sources of fluorescence within the mid-torso seemed to contribute to the signal, since the RMP and CPZ liver tissue signal was much lower than expected. To reconcile this discrepancy, we examined other tissues that could contribute to liver region imaging, including the stomach, pancreas, spleen, and abdominal fat. It was interesting to note that both RMP and CPZ mice showed increased kidney signal, which was sufficiently bright to contribute to the liver region imaging results. These results led us to two critical conclusions: 1) epifluorescence is a fast and effective screening approach, but results need to be interpreted carefully, and 2) the AMT-750 cocktail has the potential to detect not only DILI but also injury to other tissues.

#### A Comprehensive Screening Paradigm Using AMT-750 to Detect Chemically Induced Injury to Liver and Other Tissues In Vivo and Ex Vivo.

**Fig. 5.**
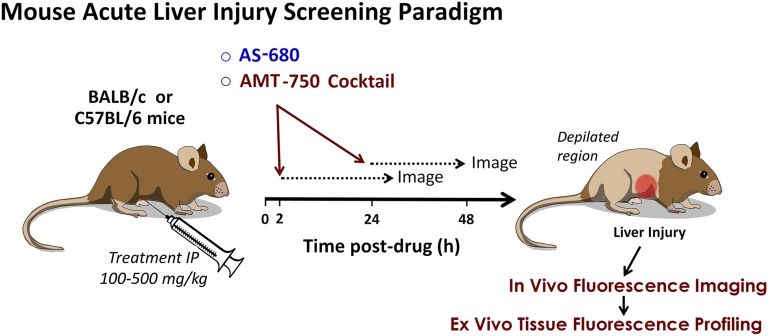
Mouse acute liver injury screening paradigm. Depilated male BALB/c or C57BL/6 mice (Charles River Laboratories) were injected intraperitoneally with DILI-inducing drugs. Separate cohorts of mice, at 2 and 18–24 hours post-treatment, were then injected with imaging probes (AMT-750 and AS-680) for imaging 24 hours later to detect biologic changes within damaged tissue. IP, intraperitoneal.

Imaging results from the studies represented in Figs. 1–4 established an effective protocol for acquiring quantification of biologic liver changes; however, it also involved extensive pilot studies to establish the right doses, conditions, and time points tailored to the agents tested. Because our goal was to establish an early drug discovery–stage screening process for assessing potential liabilities of new chemical entities, it was important to establish a testing paradigm that takes into account additional factors such as contribution from nonhepatic tissues, the impact of nonspecific accumulation, and the need to capture the right time point. In addition, interpretation of AMT-750 results can be compromised by nonspecific accumulation in tissue due to an increased vascular leak, so we included AS-680 to allow for subtraction or normalization to compensate for vascular effects. In our experience, the optimal time for injecting imaging probes (post-treatment) will generally be at either 2 hours or 24 hours, with liver injury induction producing a good liver AMT-750 signal when imaged 24 hours after at least one of these injection times. Therefore, we developed a screening paradigm (illustrated in Fig. 5) that maximizes the possibility of detecting tissue biologic changes associated with drug dosing. In addition, we broadened the approach to include assessment of other organs and tissues. The cocktail of probes is ideal for such broad tissue detection; cell death (AV-750) and inflammation (MMP-750) are obviously not limited to specific tissues, and transferrin receptors, good indicators of cellular iron metabolism (TfV-750), are expressed in a wide variety of normal tissues, including the liver, spleen, brain, lung, muscle, testes, kidney, and heart (Kawabata et al., 2001).

**Fig. 6.**
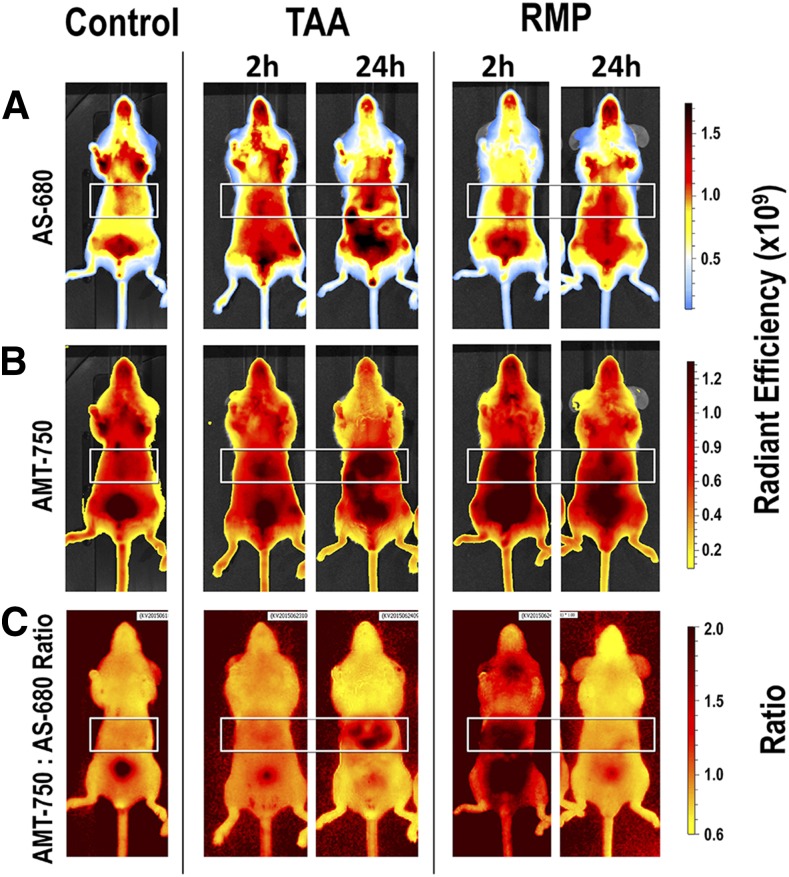
Ratio of AMT-750 to vascular leak (AS-680) to correct for nonspecific accumulation of AMT-750 in tissues. Mice (*n* = 3 per group) pretreated with TAA or RMP were coinjected with the AMT-750 cocktail and AS-680, a well established vascular leak imaging probe, to allow normalization of AMT-750 data. Whole-mouse epifluorescence imaging was used to detect accumulation of the 680-nm and 750-nm probes in the liver regions of different cohorts of mice post-treatment. (A and B) Epifluorescence images of AS-680 signal (A) and AMT-750 signal (B) (middle panel) in controls and in treated mice at two times post-treatment. (C) Normalized ratio images (AMT-750/AS-680) were generated on IVIS SpectrumCT Living Image 4.5 software. White boxes indicate the liver regions analyzed for fluorescent signal. Results are representative of three studies using probe cocktail imaging.

**Fig. 7.**
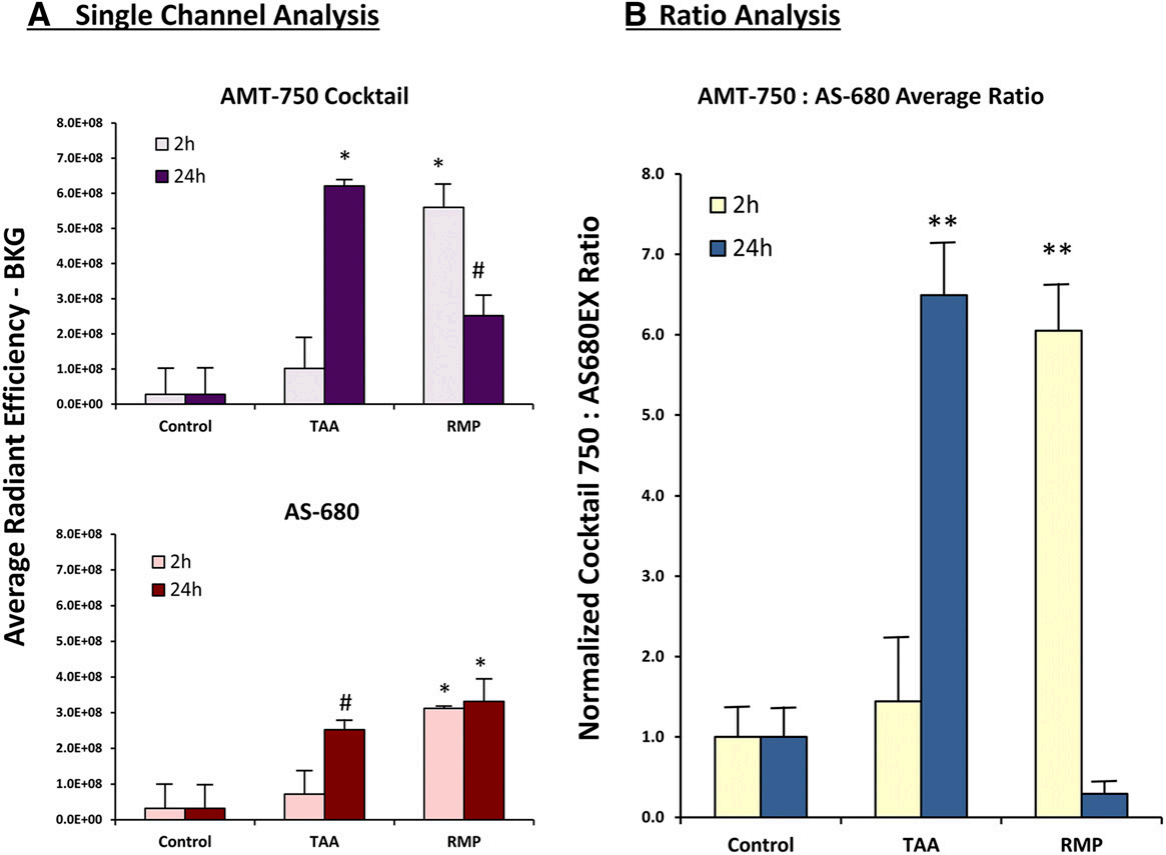
Quantification of AMT-750/AS-680 ratios for quantification of DILI. Single-channel quantification of the liver region fluorescence signal from noninvasive imaging was determined by region-of-interest placement to capture the entire liver. Results are represented with 90% of the negative control liver signal subtracted. (A) Results from the AMT-750 signal (upper panel) and the AS-680 signal (lower panel) are represented as background-corrected average total radiant efficiency. (B) Ratio analysis of AMT-750 to AS-680, using Living Image 4.5 image math functions, was performed to correct AMT-750 results for nonspecific vascular leak contribution to the overall signal. Results are expressed as ratios of AMT-750 to AS-680 subsequently normalized to set average control ratios to 1. Statistical significance was assessed by analysis of variance with the Dunnett post-test (*P < 0.01; **P < 0.001; ^#^*P* < 0.05; *n* = 3). BKG, background signal levels.

Our screening approach uses as few as six to eight mice per test compound (divided into two probe injection time points, each with three to four animals), with an additional three to four control animals that also receive AMT-750/AS-680. We focused on two of our liver injury–inducing agents, TAA and RMP, which differ in liver injury mechanism as well in the kinetics of injury. The TAA group showed minimal changes when probes were injected at 2 hours; however, the peak vascular leak and AMT-750 signal occurred when probes were injected at 24 hours and imaged at 48 hours. In contrast, the RMP group showed an early AMT-750 signal, a minimal late AMT-750 signal, and a comparable vascular leak both early and late. Two-channel imaging on the IVIS SpectrumCT allowed the use of image math functions on the system software (Living Image 4.5; PerkinElmer) to provide pixel-by-pixel ratios of AMT-750 to AS-680. Such ratio analysis allows correction of AMT750 image data for the contribution of nonspecific tissue accumulation. This gives clear corrected fluorescent images (Fig. 6) and reveals that a portion of the AMT-750 signal could be attributed to alterations in the vascular leak into the tissues in the liver region. The corrected images removed the early AMT-750 signal in TAA mice and almost completely removed the late signal in RMP mice. Quantitatively, both the AMT-750 and AS-680 probes showed statistically significant increases in the liver region signal associated with the treatments (Fig. 7). The ratio data improved the quantification of AMT-750 by removing the contribution from vascular leak and was a useful comparator to the AS-680 data alone, with the first showing corrected biologic marker change and the second showing vascular leak.

**Fig. 8.**
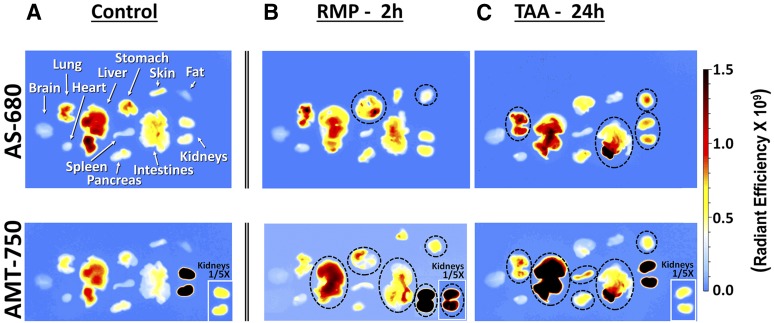
Ex vivo AMT-750 and AS-680 imaging of excised tissues from control, RMP-treated, and TAA-treated mice. (A–C) Single-channel images of excised liver, brain, lung, heart, stomach, skin, fat, spleen, pancreas, intestine, and kidney tissues were acquired for control (A), RMP treatment (B), and TAA treatment (C) with both AS-680 (upper panels) and AMT-750 (lower panels) imaging probes. Images were acquired for the optimal time points for each treatment: 2 hours for RMP and 24 hours for TAA. AMT-750 images were optimized for liver visualization, which led to saturation of the kidney signal (mostly due to known kidney clearance of AV-750). Insets provide optimized images for the kidney, revealing an enhanced signal in kidneys from RMP-treated mice.

**Fig. 9.**
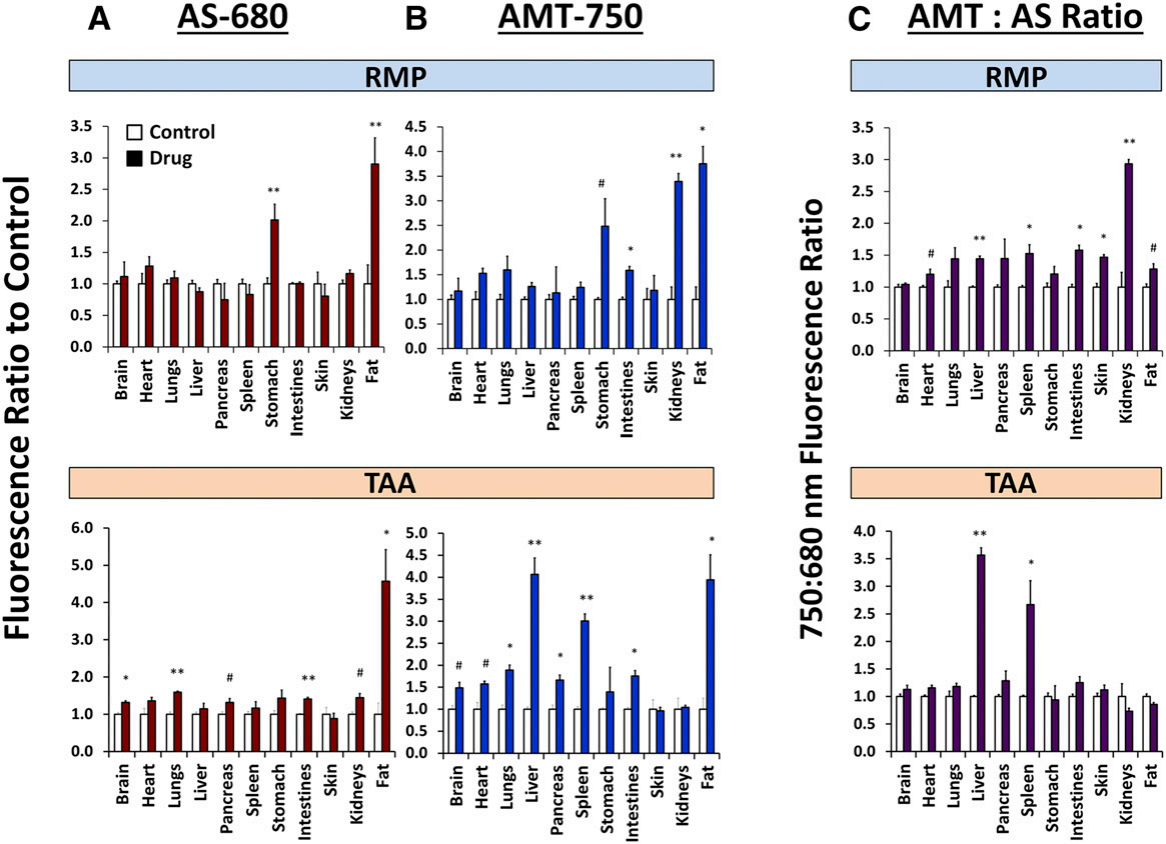
Quantification of fluorescence in excised tissues from control, RMP-treated, and TAA-treated mice. Regions of interest were placed to quantify fluorescence levels in the liver, brain, lung, heart, stomach, skin, fat, spleen, pancreas, intestine, and kidney tissues from control, TAA-treated, and RMP-treated mice that had been injected with both AS-680 and AMT-750. (A–C) AS-680 (A), AMT-750 (B), and AMT-750/AS-680 ratio (C) fluorescence datasets were quantified, and data were normalized . Results are represented as ratio to control ± S.E.M., and statistical significance was assessed by analysis of variance with the Dunnett post-test (*P < 0.01; **P < 0.001; ^#^*P* < 0.05; *n* = 3). Results are representative of multiple studies using either multiplex or individual probe imaging.

To capture additional potential chemically induced tissue effects, mice were euthanized after in vivo imaging and the brain, lung, heart, liver, stomach, skin, abdominal fat, spleen, pancreas, intestines, and kidneys were removed. Ex vivo imaging of these tissues (Fig. 8), again acquiring both AMT-750 and AS-680 images, allowed detection of biologic changes induced by chemical agent administration. For defined organs (i.e., brain, heart, lungs, liver, pancreas, spleen, stomach intestines, and kidneys), analysis was performed by measuring total fluorescence. To avoid tissue size bias in skin and abdominal fat tissue samples, analysis was performed by quantifying the fluorescence within standard-sized regions of interest placed to capture the majority of the tissue area. RMP showed some interesting changes, including stomach enlargement (and increases in both AMT-750 and AS-680 due to this enlargement), an increased signal in fat, and AMT-750 increases in intestines and kidneys. TAA showed late vascular leak changes in lung, intestines, kidneys, and fat; there were AMT-750 increases in lungs, liver, spleen, pancreas, intestines, and fat. Quantitation of these ex vivo results (Fig. 9) showed statistically significant changes in the stomach, kidneys, and abdominal fat with RMP treatment. Ratio analysis further suggested modest changes in numerous tissues, including the liver, in agreement with the generally elevated whole-body signal seen with in vivo imaging. Interestingly, and in agreement with the study represented by Fig. 4, the kidneys were much more affected by RMP than was the liver, revealing that although the signal was significantly elevated in the liver region, the overall abdominal region signal was dominated by greater increases in kidney fluorescence. In contrast, TAA induced a variety of modest vascular leak changes in a variety of tissues; however, the predominant AMT-750 changes were seen in the liver and spleen once the signal was corrected for a nonspecific vascular leak. Most of our findings, here using a single dose of chemical agent, are supported by previously published studies performed after repeated dosing of RMP (Poole et al., 1971; Bykova et al., 1977; Roszkowski et al., 1984; Katz and Lor, 1986; Zargar et al., 1990; Sodhi et al., 1997; Tassaduq et al., 2011; Chiba et al., 2013) or TAA (al-Bader et al., 2000; Wang et al., 2004).

#### Histology Assessment of Single-Dose Drug Effects on Tissues Identified by Biomarker Imaging.

**Fig. 10.**
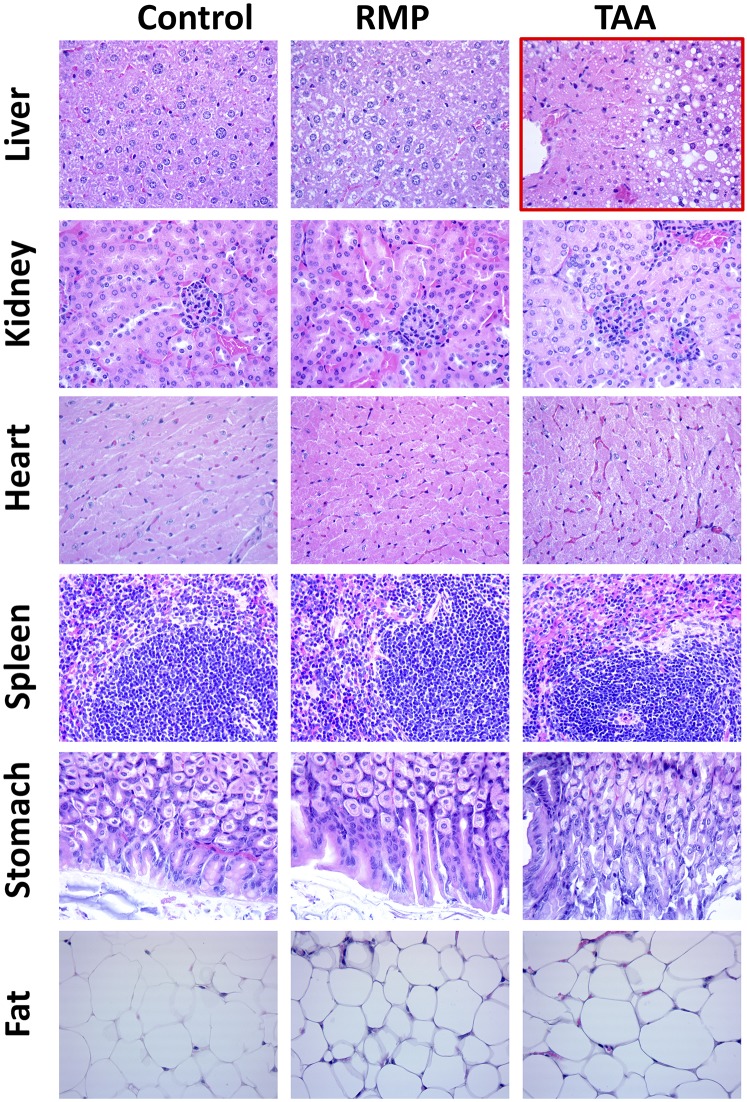
Histologic assessment of tissues from control, RMP-treated, and TAA-treated mice. Liver, kidney, heart, spleen, stomach, and fat tissues were collected from control, RMP-treated, and TAA-treated BALB/c mice. Tissues were fixed in 10% neutral buffered formalin for 24 hours at 20°C, followed by storage in 70% ethanol. Tissues were then embedded in paraffin, sectioned (4 *µ*m), and stained with hematoxylin and eosin for general evaluation of pathologic changes. Results were assessed by a pathologist, and the only gross abnormality seen across the range of tissues was moderate necrosis induced by TAA (see red-outlined image).

In vivo fluorescence imaging of RMP- and TAA-treated mice suggested that these treatments induced significant biologic changes that could be readily detected by our AMT-750 probe cocktail. Our previous studies with APAP established that even a single bolus drug dose induces obvious tissue histology changes (Peterson, 2016), and additional studies showed consistent detection of TAA- and CPZ-induced tissue damage with little or no obvious effects by RMP (data not shown). To understand the imaging results from RMP- and TAA-treated mice, in the context of overt tissue changes, we collected various tissues (liver, kidney, heart, spleen, stomach, and fat) from treated animals, and a trained pathologist assessed the hematoxylin and eosin–stained sections (Fig. 10). Liver results with TAA were reviewed by the pathologist as showing moderate necrosis after the single bolus dose of 300 mg/kg, whereas the same bolus dose of RMP induced no perceptible tissue changes. It is interesting to note that the four other sets of tissues in which we expected to see some level of tissue change—spleen (TAA), kidney (RMP), stomach (RMP), and fat (TAA and RMP)—showed no overt signs of injury. This supports the expectation that biologic changes would likely precede overt signs of tissue injury, when one considers additional published data with repeated dosing in animals and human patients (Poole et al., 1971; Roszkowski et al., 1984; Katz and Lor, 1986; Zargar et al., 1990; al-Bader et al., 2000; Wang et al., 2004; Tassaduq et al., 2011; Kadir et al., 2013).

## Discussion

In vivo preclinical toxicology studies use living animals to assess potential drug-induced adverse effects, providing models in which potential toxicity is driven by the combined effects of drug distribution/metabolism, drug mechanistic efficacy, and the complex biology and physiology of a living animal. However, this complexity in animals may not always align completely with the complexity in humans, so there is always concern regarding the capability of preclinical toxicology assessment to predict clinical findings in humans. Clinical trials provide only a limited source of information regarding this “predictivity” because they are designed specifically to avoid adverse outcomes seen in animal studies. Rather, preclinical testing allows the establishment of a risk assessment strategy in which the worst of the tested chemical compounds are considered too risky and never administered to humans, those with some adverse findings are used with caution and guidance from preclinical studies, and compounds with no apparent toxicity are progressed with healthy skepticism. It is in this way that preclinical toxicity testing of small- and large-molecule pharmaceuticals can play a valuable informative role in drug discovery and development; however, this information often comes late in the discovery process. Generally, formal toxicology studies are large resource investments, predominantly employed at the late stages of drug discovery, and are performed for advanced candidates to support regulatory filings. However, under this paradigm, the high failure rate of new drugs, both in preclinical development and in clinical trials (Ahuja and Sharma, 2014), suggests a need for both better selection criteria for development candidates and earlier elimination of toxic compounds prior to selection as development candidates. Sensitive toxicology screening assays are needed that: 1) are designed for use earlier in the drug discovery process, during early preclinical lead selection; 2) are medium to high throughput to allow parallel assessment of multiple compounds; 3) emphasize the sensitive detection of biologic, rather than gross, changes in tissue or in plasma biomarkers; and 4) offer the potential for broader assessment of additional nonhepatic tissues. Preclinical biologic imaging strategies may be uniquely suited to addressing these needs.

NIR fluorescence preclinical imaging offers a useful approach to detect in situ dynamic changes in tissue biology through the use of highly stable, targeted, vascular, or protease-activated imaging probes. When considered in the context of either disease or toxicology/safety, imaging brings an aspect of noninvasive assessment of biologic changes rather than gross physiologic or ex vivo morphologic changes. Imaging probes are designed to detect tissue biomarkers and accumulate directly at the local site of tissue injury, offering rapid multiplexing imaging of various biologic readouts. NIR fluorescent probes have been used effectively to image various disease conditions modeled in preclinical species, including cancer (Weissleder and Ntziachristos, 2003; Montet et al., 2005; Kossodo et al., 2010; Bao et al., 2012), atherosclerosis (Chen et al., 2002; Deguchi et al., 2006), cardiac pathologies (Nahrendorf et al., 2007; Sosnovik et al., 2007), arthritis (Wunder et al., 2004; Peterson et al., 2010), central nervous system demyelination (Eaton et al., 2013), and osteoarthritis (Vermeij et al., 2014). Many of these same fluorescent imaging probes are currently being examined for their utility in toxicology/safety screening (Peterson, 2016). Our current studies show this to be a valuable approach, because some of the same biologic/physiologic processes and cellular players involved in various types of disease progression are also associated with drug-induced toxicity. To this end, we established an in vivo early screening liver injury protocol that is simple and fast, relying on a single bolus dose of test compounds. A cocktail of three different NIR fluorescent imaging probes—AV-750 (cell death), MMP-750 (inflammation), and TfV-750 (metabolism)—detects the effects of single bolus intraperitoneal doses of four different chemical agents known to induce liver injury by either hepatocellular, cholestatic, or mixed mechanisms. This technique provides robust quantitative analysis, as well as the ability to assess DILI noninvasively in vivo. The probe cocktail has the added advantage of also detecting damage occurring in a variety of other tissues, including the kidney, spleen, and fat, further broadening the utility of the assessment (see Figs. 8 and 9). This was a surprising finding for this probe cocktail, and it is currently under study to further understand its utility as a broad screening tool for whole-body drug-induced tissue injury.

It is important to consider the practical application of our NIR fluorescence imaging approach for very early liver injury screening. Any effective approach needs to address mechanistic tissue injury kinetics, and we have found that some compounds alter tissue biology within the first several hours after administration, whereas others require at least 24 hours. This is why our recommended protocol includes injecting the AMT-750 cocktail at both 2 hours and 24 hours in separate cohorts (for imaging at 26 hours and 48 hours, respectively) to minimize the risk of an experimentally biased false negative result. Any toxicology/safety screening protocol also needs to take into account dose dependence but also must provide a quick, practical assessment. To this end, we recommend using a single high-dose assessment of compounds at the highest tolerated dose, as well as the simultaneous injection of AS-680 with the AMT-750 cocktail. This dual imaging approach provides the maximal chance to detect liver changes and can also separate vascular-damaging effects from tissue effects (discussed further below). It has been our approach to rescreen compounds at a lower dose if the high dose shows predominantly vascular damage, although this has mostly been to cleanly assess the utility of the AMT-750 cocktail without the complication of increased passive leakage into tissues. The absolute dose levels for testing may remain somewhat arbitrary (e.g., we set this as 300 mg/kg), as we envision these studies being performed as intraperitoneal injections in testing early lead compound series, prior to establishing efficacious doses and prior to structure-activity relationship improvement of efficacy. In addition, researchers should also consider the assessment of both male and female mice if there are anticipated sex differences in drug-induced tissue injury or if there is an interest in assessing specific changes in signal in sexual organs. It is also our expectation that this imaging strategy could identify risky compounds for which the researcher can adopt a strategy of either: 1) prioritization of compounds versus by in vivo risk assessment or 2) follow-up with additional conventional testing. An additional strategy would be to use this approach to follow a series of compounds through the discovery process as “spot checks” on adverse tissue effects, perhaps adopting dosage levels relative to efficacious doses.

It is also important to consider in vivo screening specificity as well as the relevance and utility of ex vivo tissue/organ imaging. The ability of the AMT-750 cocktail to detect changes even in nonhepatic tissues (applied to four chemical agents with well defined injury in the liver and other tissues) gave us the means to validate our imaging protocol. Our findings generally aligned well with known patterns of tissue injury: 1) detailed ex vivo tissue profiling of TAA showed effects in the liver as well as the spleen, in agreement with published studies; and 2) RMP showed effects predominantly in the kidneys and stomach, with broad effects on a number of other tissues, including the liver, also in agreement with published studies. Normalization of AMT-750 results to AS-680 vascular imaging appears to offer some additional benefits in compensating for nonspecific tissue accumulation due to vascular leak (Figs. 6 and 7). Histology assessment for such acute treatments did not detect gross tissue changes, except for TAA effects on the liver; however, it is our experience that only hepatocellular toxicity–inducing treatments (including APAP, data not shown) show acute histologic changes, whereas the more subtle biologic changes associated with cholestasis may take much longer to manifest at the tissue level. With regard to the accuracy of noninvasive in vivo imaging, the screening of supine, depilated mice by 2D fluorescence is highly effective in detecting drug-induced liver effects in most cases. However, there are some occasions with supine animal positioning in which a high kidney signal, expected with RMP and CPZ (Berg and Bergan, 1976; Cuche et al., 1982; Katz and Lor, 1986), can be detected (see Fig. 4). This is only a confounding issue if the researcher does not perform ex vivo imaging of tissues after in vivo imaging. Doing both supine and prone imaging can help to provide proper interpretation of drug effects, and tomographic imaging (although lower throughput) can accurately separate liver and kidney fluorescence in one scan.

Of course, there are no preclinical animal assessments that can exclude the possibility of false negative results as compared with results in humans, which would likely be due to species differences in metabolism or immune responses. In particular, idiosyncratic drug reactions (IDRs), although not the most common type of adverse drug reaction, occur in few patients and are difficult to model or predict preclinically. The lack of clear drug dose dependence and the association with delayed onset has generally suggested that IDRs are due to induced immunologic responses. However, there are a number of manifestations apparently involving numerous potential mechanisms, including systemic hematologic changes, generalized or organ/tissue-specific autoimmunity, and rashes driven by T cells, neutrophils, or eosinophils. The strength of this imaging approach, however, is the fact that it sensitively detects drug-induced biologic changes in tissues often in the absence of overt tissue morphology/phenotype changes (Fig. 10). In recent studies, we have used the AMT-750 cocktail to detect apparent 5-fluorouracil–induced heart changes, in the absence of tissue pathology, that may be indicative of idiosyncratic 5-fluorouracil–induced cardiotoxicity (data not shown). In addition, the MMP activity detected by this cocktail could be a useful biomarker for neutrophilia or eosinophilia associated with some forms of IDR. Other forms of IDR, however, are associated with cognate immunity (i.e., T cell activation and function) and may not be amenable to detection by AMT-750. Ongoing efforts with probes currently in development for the assessment of immune function may yield tools for assessing more complex forms of immune-driven IDRs in repeat dosing preclinical rodent models.

It is important to note that optical imaging does not need to provide a solution for every toxicology question, as there are a variety of currently validated approaches that cover these important needs. Clearly, more work and more validation using a range of toxic compounds is essential to better understand the potential for false positive and false negative results. It is our belief that there may be instances in which optical imaging can provide superior readouts, equivalent readouts with easier performance, or even qualitative readouts with greater efficiency and consistency. With small numbers of animals, small quantities of test compound, and a small amount of imaging probes, results can be obtained in vivo and ex vivo within just a couple of days. The additional benefit of quick and easy detection of extrahepatic tissue injury, from the same animals and with the same imaging probes, further strengthens the utility of the approach. Our current belief is that the best fit for optical imaging is in surrogate early risk assessment screening of lead compounds, limiting the amounts of test materials required and facilitating internal decisions with regard to which lead compounds are best suited for optimization. The final assessment of regulatory-enabling toxicology studies will likely continue to be performed by more conventional means in rats and larger appropriate species, but perhaps the outcomes of these conventional assays will be more favorable for regulatory filing with the prior removal of high-risk compounds early in the discovery process.

## Abbreviations

2D: two-dimensional
3D: three-dimensional
ALP: alkaline phosphatase
ALT: alanine transaminase
APAP: acetaminophen
AS-680: AngioSense 680
AV-750: Annexin-Vivo 750
CPZ: chlorpromazine
DILI: drug-induced liver injury
IDR: idiosyncratic drug reaction
MMP: matrix metalloprotease
MMP-645: MMPSense 645
MMP-750: MMPSense 750
NIR: near-infrared
RMP: rifampicin
TAA: thioacetamide
TfV-750: Transferrin-Vivo 750

## References

[B1] Ackerman Z Pappo O Link G Glazer M Grozovski M (2015) Liver toxicity of thioacetamide is increased by hepatocellular iron overload. Biol Trace Elem Res 163:169–176. 25161090 10.1007/s12011-014-0110-9

[B2] Ahuja V Sharma S (2014) Drug safety testing paradigm, current progress and future challenges: an overview. J Appl Toxicol 34:576–594. 24777877 10.1002/jat.2935

[B3] al-Bader A Mathew TC Khoursheed M Asfar S al-Sayer H Dashti HM (2000) Thioacetamide toxicity and the spleen: histological and biochemical analysis. Anat Histol Embryol 29:3–8. 10820895 10.1046/j.1439-0264.2000.00207.x

[B4] Al-Olayan EM El-Khadragy MF Aref AM Othman MS Kassab RB Abdel Moneim AE (2014) The potential protective effect of Physalis peruviana L. against carbon tetrachloride-induced hepatotoxicity in rats is mediated by suppression of oxidative stress and downregulation of MMP-9 expression. Oxid Med Cell Longev 2014:381413. 24876910 10.1155/2014/381413 PMC4020166

[B5] Amacher DE Adler R Herath A Townsend RR (2005) Use of proteomic methods to identify serum biomarkers associated with rat liver toxicity or hypertrophy. Clin Chem 51:1796–1803. 16099942 10.1373/clinchem.2005.049908

[B6] Amoozegar CB Wang T Bouchard MB McCaslin AF Blaner WS Levenson RM Hillman EM (2012) Dynamic contrast-enhanced optical imaging of in vivo organ function. J Biomed Opt 17:96003-1. 23085904 10.1117/1.JBO.17.9.096003 PMC3434471

[B7] Anderson ER Shah YM (2013) Iron homeostasis in the liver. Compr Physiol 3:315–330. 23720289 10.1002/cphy.c120016 PMC3936199

[B8] Bao B Groves K Zhang J Handy E Kennedy P Cuneo G Supuran CT Yared W Rajopadhye M Peterson JD (2012) In vivo imaging and quantification of carbonic anhydrase IX expression as an endogenous biomarker of tumor hypoxia. PLoS One 7:e50860. 23226406 10.1371/journal.pone.0050860 PMC3511310

[B9] Berg KJ Bergan A (1976) Effects of different doses of chlorpromazine on renal function in the dog. Scand J Clin Lab Invest 36:787–794. 1031491 10.3109/00365517609081938

[B10] Bhattacharya S Zhang Q Carmichael PL Boekelheide K Andersen ME (2011) Toxicity testing in the 21 century: defining new risk assessment approaches based on perturbation of intracellular toxicity pathways. PLoS One 6:e20887. 21701582 10.1371/journal.pone.0020887 PMC3118802

[B11] Bissell DM Gores GJ Laskin DL Hoofnagle JH (2001) Drug-induced liver injury: mechanisms and test systems. Hepatology 33:1009–1013. 11283870 10.1053/jhep.2001.23505

[B12] Bykova MA Solov’ev VN Berezhinskaia VV Egorenko GG Firsov AA (1977) [Pharmacology of rifampicin]. Antibiotiki 22:525–530. 883799

[B13] Cairo G Pietrangelo A (1995) Nitric-oxide-mediated activation of iron-regulatory protein controls hepatic iron metabolism during acute inflammation. Eur J Biochem 232:358–363. 7556182 10.1111/j.1432-1033.1995.358zz.x

[B14] Chen J Tung CH Mahmood U Ntziachristos V Gyurko R Fishman MC Huang PL Weissleder R (2002) In vivo imaging of proteolytic activity in atherosclerosis. Circulation 105:2766–2771. 12057992 10.1161/01.cir.0000017860.20619.23

[B15] Chiba S Tsuchiya K Sakashita H Ito E Inase N (2013) Rifampicin-induced acute kidney injury during the initial treatment for pulmonary tuberculosis: a case report and literature review. Intern Med 52:2457–2460. 24190152 10.2169/internalmedicine.52.0634

[B16] Cuche JL Prinseau J Baglin A Guédon J (1982) [Renal effects of chlorpromazine in dogs]. Nephrologie 3:111–115. 7144993

[B17] Daghighi S, Sjollema J, Dijkstra RJ, Jaspers V, Zaat SA, van der Mei HC, and Busscher HJ (2014) Real-time quantification of matrix metalloproteinase and integrin alphavbeta3 expression during biomaterial-associated infection in a murine model. *Eur Cell Mater.* 27:26–37; discussion 37–38. 10.22203/ecm.v027a03 24464726

[B18] Deguchi JO Aikawa M Tung CH Aikawa E Kim DE Ntziachristos V Weissleder R Libby P (2006) Inflammation in atherosclerosis: visualizing matrix metalloproteinase action in macrophages in vivo. Circulation 114:55–62. 16801460 10.1161/CIRCULATIONAHA.106.619056

[B19] Domitrović R Jakovac H Marchesi VV Blažeković B (2013) Resolution of liver fibrosis by isoquinoline alkaloid berberine in CCl_4_-intoxicated mice is mediated by suppression of oxidative stress and upregulation of MMP-2 expression. J Med Food 16:518–528. 23734997 10.1089/jmf.2012.0175 PMC3684211

[B20] Duan L Davis JS Woolbright BL Du K Cahkraborty M Weemhoff J Jaeschke H Bourdi M (2016) Differential susceptibility to acetaminophen-induced liver injury in sub-strains of C57BL/6 mice: 6N versus 6J. Food Chem Toxicol 98 (Pt B):107–118. 27773698 10.1016/j.fct.2016.10.021 PMC5123947

[B21] Eaton VL Vasquez KO Goings GE Hunter ZN Peterson JD Miller SD (2013) Optical tomographic imaging of near infrared imaging agents quantifies disease severity and immunomodulation of experimental autoimmune encephalomyelitis in vivo. J Neuroinflammation 10:138. 24237884 10.1186/1742-2094-10-138 PMC4225609

[B22] Gibb S (2008) Toxicity testing in the 21st century: a vision and a strategy. Reprod Toxicol 25:136–138. 18093799 10.1016/j.reprotox.2007.10.013

[B23] Ging P Mikulich O O’Reilly KM (2016) Unexpected paracetamol (acetaminophen) hepatotoxicity at standard dosage in two older patients: time to rethink 1 g four times daily? Age Ageing 45:566–567. 27103600 10.1093/ageing/afw067

[B24] Izawa T Murakami H Wijesundera KK Golbar HM Kuwamura M Yamate J (2014) Inflammatory regulation of iron metabolism during thioacetamide-induced acute liver injury in rats. *Exp Toxicol Pathol* 66:155–162. 10.1016/j.etp.2013.12.002 24373749

[B25] Kadir FA Kassim NM Abdulla MA Yehye WA (2013) Effect of oral administration of ethanolic extract of Vitex negundo on thioacetamide-induced nephrotoxicity in rats. BMC Complement Altern Med 13:294. 24499255 10.1186/1472-6882-13-294 PMC4028978

[B26] Katz MD Lor E (1986) Acute interstitial nephritis associated with intermittent rifampin use. Drug Intell Clin Pharm 20:789–792. 3769771 10.1177/106002808602001014

[B27] Kawabata H Germain RS Ikezoe T Tong X Green EM Gombart AF Koeffler HP (2001) Regulation of expression of murine transferrin receptor 2. Blood 98:1949–1954. 11535534 10.1182/blood.v98.6.1949

[B28] Korideck H Peterson JD (2009) Noninvasive quantitative tomography of the therapeutic response to dexamethasone in ovalbumin-induced murine asthma. J Pharmacol Exp Ther 329:882–889. 19293392 10.1124/jpet.108.147579

[B29] Kossodo S Pickarski M Lin SA Gleason A Gaspar R Buono C Ho G Blusztajn A Cuneo G Zhang J, et al. (2010) Dual in vivo quantification of integrin-targeted and protease-activated agents in cancer using fluorescence molecular tomography (FMT). *Mol Imaging Biol* 12:488–499. 10.1007/s11307-009-0279-z 19960268

[B30] Krautz-Peterson G Ndegwa D Vasquez K Korideck H Zhang J Peterson JD Skelly PJ (2009) Imaging schistosomes in vivo. FASEB J 23:2673–2680. 19346298 10.1096/fj.08-127738 PMC2717771

[B31] Krewski D Westphal M Al-Zoughool M Croteau MC Andersen ME (2011) New directions in toxicity testing. Annu Rev Public Health 32:161–178. 21219154 10.1146/annurev-publhealth-031210-101153

[B32] Lin SA Patel M Suresch D Connolly B Bao B Groves K Rajopadhye M Peterson JD Klimas M Sur C (2012) Quantitative longitudinal imaging of vascular inflammation and treatment by ezetimibe in apoE mice by FMT using new optical imaging biomarkers of cathepsin activity and α(v)β(3) integrin. Int J Mol Imaging 2012:189254. 23119157 10.1155/2012/189254 PMC3483711

[B33] Maddox JF Amuzie CJ Li M Newport SW Sparkenbaugh E Cuff CF Pestka JJ Cantor GH Roth RA Ganey PE (2010) Bacterial- and viral-induced inflammation increases sensitivity to acetaminophen hepatotoxicity. J Toxicol Environ Health A 73:58–73. 19953420 10.1080/15287390903249057

[B34] McGill MR Williams CD Xie Y Ramachandran A Jaeschke H (2012) Acetaminophen-induced liver injury in rats and mice: comparison of protein adducts, mitochondrial dysfunction, and oxidative stress in the mechanism of toxicity. Toxicol Appl Pharmacol 264:387–394. 22980195 10.1016/j.taap.2012.08.015 PMC3478469

[B35] Montet X Ntziachristos V Grimm J Weissleder R (2005) Tomographic fluorescence mapping of tumor targets. Cancer Res 65:6330–6336. 16024635 10.1158/0008-5472.CAN-05-0382

[B36] Mullock BM Hall DE Shaw LJ Hinton RH (1983) Immune responses to chlorpromazine in rats. Detection and relation to hepatotoxicity. Biochem Pharmacol 32:2733–2738. 6626244 10.1016/0006-2952(83)90084-9

[B37] Nahrendorf M Swirski FK Aikawa E Stangenberg L Wurdinger T Figueiredo JL Libby P Weissleder R Pittet MJ (2007) The healing myocardium sequentially mobilizes two monocyte subsets with divergent and complementary functions. J Exp Med 204:3037–3047. 18025128 10.1084/jem.20070885 PMC2118517

[B38] Peterson JD (2016) Noninvasive in vivo optical imaging models for safety and toxicity testing, in Nutraceuticals: Efficacy, Safety and Toxicity (Gupta RC ed) pp 305–317, Academic Press, London.

[B39] Peterson JD Labranche TP Vasquez KO Kossodo S Melton M Rader R Listello JT Abrams MA Misko TP (2010) Optical tomographic imaging discriminates between disease-modifying anti-rheumatic drug (DMARD) and non-DMARD efficacy in collagen antibody-induced arthritis. Arthritis Res Ther 12:R105. 20509880 10.1186/ar3038 PMC2911895

[B40] Poole G Stradling P Worlledge S (1971) Potentially serious side-effects of high-dose twice-weekly rifampicin. Postgrad Med J 47:727–747. 5159676 10.1136/pgmj.47.553.742 PMC2467359

[B41] Roszkowski W Lipinska R Roszkowski K Jeljaszewicz J Pulverer G (1984) Rifampicin-induced suppression of antitumor immunity. Med Microbiol Immunol (Berl) 172:197–205. 6717373 10.1007/BF02123714

[B42] Shuhendler AJ Pu K Cui L Uetrecht JP Rao J (2014) Real-time imaging of oxidative and nitrosative stress in the liver of live animals for drug-toxicity testing. Nat Biotechnol 32:373–380. 24658645 10.1038/nbt.2838 PMC4070437

[B43] Sodhi CP Rana SV Mehta SK Vaiphei K Attari S Mehta S (1997) Study of oxidative-stress in isoniazid-rifampicin induced hepatic injury in young rats. Drug Chem Toxicol 20:255–269. 9292280 10.3109/01480549709003881

[B44] Sosnovik DE Nahrendorf M Weissleder R (2007) Molecular magnetic resonance imaging in cardiovascular medicine. Circulation 115:2076–2086. 17438163 10.1161/CIRCULATIONAHA.106.658930

[B45] Tassaduq I Butt SA Hamid S (2011) Protective effect of ascorbic acid on rifampicin induced hepatotoxicity in mice. J Rawalpiindi Med Coll 15:102–103.

[B46] Vermeij EA Koenders MI Blom AB Arntz OJ Bennink MB van den Berg WB van Lent PL van de Loo FA (2014) In vivo molecular imaging of cathepsin and matrix metalloproteinase activity discriminates between arthritic and osteoarthritic processes in mice. Mol Imaging 13:1–10. 24881106

[B47] Wang CH Chen YJ Lee TH Chen YS Jawan B Hung KS Lu CN Liu JK (2004) Protective effect of MDL28170 against thioacetamide-induced acute liver failure in mice. J Biomed Sci 11:571–578. 15316131 10.1007/BF02256121

[B48] Weissleder R Ntziachristos V (2003) Shedding light onto live molecular targets. Nat Med 9:123–128. 12514725 10.1038/nm0103-123

[B49] Wunder A Tung CH Müller-Ladner U Weissleder R Mahmood U (2004) In vivo imaging of protease activity in arthritis: a novel approach for monitoring treatment response. Arthritis Rheum 50:2459–2465. 15334458 10.1002/art.20379

[B50] Yoon E Babar A Choudhary M Kutner M Pyrsopoulos N (2016) Acetaminophen-induced hepatotoxicity: a comprehensive update. J Clin Transl Hepatol 4:131–142. 27350943 10.14218/JCTH.2015.00052 PMC4913076

[B51] Zargar SA Thapa BR Sahni A Mehta S (1990) Rifampicin-induced upper gastrointestinal bleeding. Postgrad Med J 66:310–311. 2385557 10.1136/pgmj.66.774.310 PMC2429401

[B52] Zhang J Preda DV Vasquez KO Morin J Delaney J Bao B Percival MD Xu D McKay D Klimas M (2012) A fluorogenic near-infrared imaging agent for quantifying plasma and local tissue renin activity in vivo and ex vivo. Am J Physiol Renal Physiol 303:F593–F603. 22674025 10.1152/ajprenal.00361.2011 PMC3423114

[B53] Zhao P Zheng M Luo Z Gong P Gao G Sheng Z Zheng C Ma Y Cai L (2015) NIR-driven smart theranostic nanomedicine for on-demand drug release and synergistic antitumour therapy. Sci Rep 5:14258. 26400780 10.1038/srep14258 PMC4585834

[B54] Zítková L Stastná J Dobrovský K Tousek J (1982) Causes for rifampicin toxicity--experimental study. Czech Med 5:210–217. 6818008

